# Nephrotic Syndrome due to Focal Segmental Glomerulosclerosis Complicating Sjögren's Syndrome: A Case Report and Literature Review

**DOI:** 10.1155/2019/1749795

**Published:** 2019-08-15

**Authors:** Shigekazu Kurihara, Makoto Harada, Tohru Ichikawa, Takashi Ehara, Mamoru Kobayashi

**Affiliations:** ^1^Department of Nephrology, Nagano Red Cross Hospital, 5-22-1 Wakasato, Nagano 380-8582, Japan; ^2^Department of Nephrology, Shinshu University School of Medicine, 3-1-1 Asahi, Matsumoto 390-8621, Japan; ^3^Graduate School of Health Sciences, Matsumoto University, 2095-1 Niimura, Matsumoto 390-1241, Japan

## Abstract

**Background:**

Renal tubular acidosis and tubulointerstitial nephritis constitute the primary renal complications associated with Sjögren's syndrome (SjS), and glomerulonephritis and nephrotic syndrome are rare.

**Case Presentation:**

A 79-year-old Japanese woman presented with bilateral leg edema and weight gain and was diagnosed with nephrotic syndrome. In addition, she reported a 5-year history of dryness of mouth and was diagnosed with SjS. Renal biopsy revealed segmental glomerulosclerosis, with some specimens showing collapse of the glomerular capillary loops, proliferation of glomerular epithelial cells, and sclerotic lesions at the tubular poles, without spike formation, double contour lesions, or any other changes of the glomerular basement membrane. Immunofluorescence staining showed no immune complex (immunoglobulin IgG, IgA, or IgM) or complement (C3) deposition in the glomerular capillary walls. Based on these findings, she was diagnosed with focal segmental glomerulosclerosis (FSGS). The administration of steroid and cyclosporine achieved complete remission of nephrotic syndrome.

**Conclusion:**

Although glomerular diseases are rare, a variety of glomerular lesions including FSGS are reported in patients with SjS. Therefore, renal biopsy is warranted in patients with SjS presenting with severe urinary abnormalities.

## 1. Introduction

Sjögren's syndrome (SjS) is a complicated chronic systemic inflammatory disorder of the exocrine glands. This autoimmune disease is characterized by sialadenitis, dacryoadenitis, hypergammaglobulinemia, and positive results with tests for various autoantibodies [1]. Tubulointerstitial nephritis (TIN) and renal tubular acidosis constitute the typical renal complications associated with SjS. Glomerular diseases presenting with nephrotic syndrome are extremely rare in these patients [2, 3]. Reportedly, a variety of glomerular lesions occur in patients with SjS [4]. We describe the case of an elderly woman with nephrotic syndrome secondary to focal segmental glomerulosclerosis (FSGS) complicating SjS. Owing to the rarity of the condition, we have described the clinical course and histopathological findings in detail. In addition, we have discussed various glomerular diseases complicating SjS.

## 2. Case Report

A 79-year-old Japanese woman presented with bilateral leg edema and reported a weight gain of 7 kg over a month before admission. Urinary examination revealed increased urinary protein levels, and blood tests revealed hypoalbuminemia; thus, she was diagnosed with nephrotic syndrome. She reported a history of liver disorder diagnosed 10 years earlier along with an approximately 5-year history of dryness of mouth. Physical examination revealed attenuated breath sounds in the left lower lung field without pulmonary adventitious sounds. Examination of the bilateral lower extremities revealed pitting edema. Laboratory data are shown in Table 1. Urinary examination revealed urinary protein levels of 5711 mg/day, hematuria (20–29 red blood cells/high-power field), and urinary *N*-acetyl-beta-D-glucosaminidase and *β*2-microglobulin levels were elevated at 129.3 U/L and 2539 U/L, respectively. Blood tests revealed the following results: total protein 6.3 g/dL, serum albumin 1.7 g/dL, blood urea nitrogen 14.9 mg/dL, creatinine 0.65 mg/dL, aspartate aminotransferase 65 U/L, alanine aminotransferase 33 U/L, lactate dehydrogenase 392 U/L, alkaline phosphatase 1235 U/L, and gamma-glutamyltransferase 664 U/L. Anti-SS-A and anti-SS-B antibody test results were negative; however, the salivary glands showed reduced secretory activity, and the Saxon test results were positive. The Schirmer test also showed a positive test result. A lip biopsy revealed lymphocytic infiltration. Tests for anti-nuclear antibody and rheumatoid factor showed positive results. Salivary gland scintigraphy was not performed. Based on the American College of Rheumatology criteria determined from Sjögren International Collaborative Clinical Alliance data, she was diagnosed with SjS [5, 6]. In addition, her hepatobiliary enzyme level was elevated. Although a liver biopsy was not performed, she was clinically diagnosed with primary biliary cholangitis (PBC) based on her chronic liver disorder and positive results with anti-mitochondrial M2 antibody testing [7].

**Figure 1:**
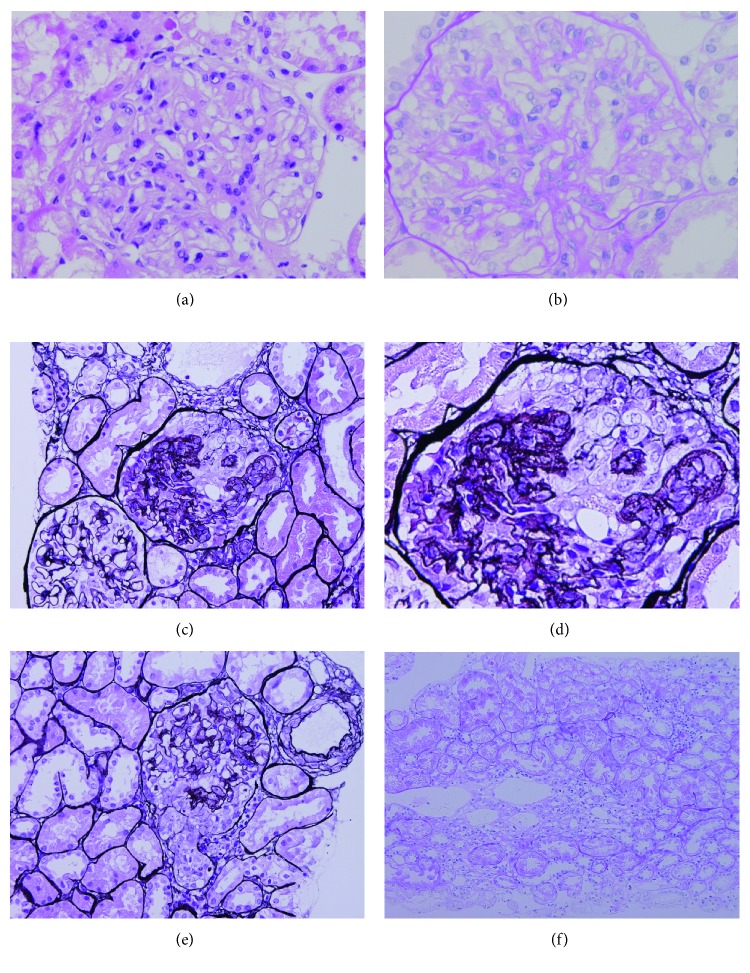
Histopathological findings in a kidney biopsy specimen showing collapse of capillary loop and segmental sclerosis with proliferation of glomerular epithelial cells. (a) Hematoxylin-eosin staining, high magnification. Intraluminal cell proliferation or necrotizing crescentic formation is not shown. (b) Periodic acid-Schiff staining, high magnification. Mesangial matrix proliferation or mesangial cell proliferation is not shown. (c) Periodic acid-methenamine-silver staining, low magnification. (d) Periodic acid-methenamine-silver staining, high magnification. A segmental lesion is observed at the tubular pole. (e) Periodic acid-methenamine-silver staining, low magnification. (f) Periodic acid-Schiff staining. Although there was slight lymphocytic infiltration in the tubulointerstitial area, obvious tubulointerstitial nephritis was not detected.

A kidney biopsy was performed, and the specimen included 33 glomeruli, with 1 showing global sclerosis and 3 showing segmental glomerulosclerosis, with a few showing collapse of the glomerular capillary loops, proliferation of glomerular epithelial cells, and sclerotic lesions identified at the tubular poles. Spike or double contour lesions were not detected in the glomerular basement membrane. Notably, histopathological findings of TIN were not detected in the specimens (Figure 1). There was one glomerulus in the immunofluorescence specimen. Immunofluorescence staining showed no glomerular deposition of immunoglobulin (IgG, IgA, and IgM), C3, or fibrinogen. Based on these findings, she was diagnosed with FSGS. Unfortunately, the specimen used for electron microscopy did not include any glomeruli. The current patients did not show any electrolyte disturbances in the blood or excess electrolyte excretion in the urine. The blood pH level of the patient did not show the acidemia. In addition, the ability to concentrate urine was normal. Concerning the pathological findings of the tubular interstitial area, it was only slight lymphocytic infiltration, and the patient did not develop obvious tubulointerstitial nephritis. Unfortunately, urinary amino acid analysis was not conducted, or titratable acid levels were not evaluated. According to these results, we considered that the current case did not have the clinical manifestations of renal tubular disease.

**Figure 2:**
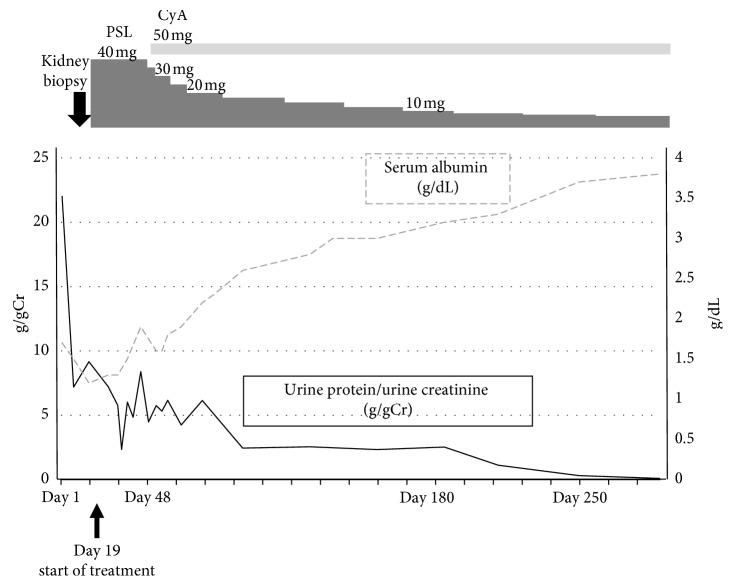
Patient's clinical course. The patient was treated with PSL and CyA. The urine protein/urine creatinine ratio gradually decreased until complete remission (urine protein excretion <0.3 g/gCr) was achieved. CyA: cyclosporine; PSL: prednisolone.

Steroid therapy was initiated (oral prednisolone at a dose of 40 mg/day). Four weeks after initiation of steroid therapy, the proteinuria was not sufficiently decreased; hence, we added cyclosporine therapy (oral dose at 50 mg/day). Her blood cyclosporine level 2 hours after the oral administration of cyclosporine was controlled at 800 ng/mL. Angiotensin II receptor blocker (irbesartan) was added to her regimen after which her urinary protein level gradually decreased with an increase in serum albumin levels. Complete remission was achieved on day 269 after treatment initiation. The urinary protein/urinary creatinine ratio was 0.09 g/g Cr, her serum albumin level increased to 3.7 g/dL, and her leg edema disappeared (Figure 2).

## 3. Discussion

In the current case, the patient developed nephrotic syndrome over the clinical course of SjS and PBC. Glomerular diseases rarely complicate SjS. Goules et al. and Maripuri et al. have reported only 5 cases of severe or nephrotic-range proteinuria in 60 patients with SjS in whom a kidney biopsy was performed [2, 3] allowing for the detection of glomerular lesions. Notably, 9 previous studies have reported renal biopsy findings in patients with SjS [2, 3, 5–11].  Table 2 shows a summary of these studies. The incidence rate of TIN was high at 37.1 to 97.9%, and glomerular diseases were rare. Glomerular diseases primarily included mesangial proliferative glomerulonephritis (including IgA nephropathy), membranous nephropathy, membranoproliferative glomerulonephritis (MPGN), FSGS, and minimal change disease. The incidence rate of FSGS was reportedly 1.6 to 8.3%.

Reportedly, renal amyloidosis [12], podocytic infolding glomerulopathy [13], lupus-associated lesions [4], and anti-neutrophil cytoplasmic antibody-associated nephritis are known to occur in patients with SjS [4]. Treatment for renal involvement primarily includes the administration of corticosteroid, and a few patients receive other immunosuppressants such as cyclosporine, cyclophosphamide, mycophenolate mofetil, and rituximab. Limited data are available regarding renal prognosis in these patients. Jasiek et al. and Ren et al. reported that 5 cases of 95 patients and 4 cases of 130 patients developed end-stage renal disease, respectively [7, 10]. There is lack of data comparing renal prognosis based on the histopathological findings of renal biopsy specimens. Notably, Kidder et al. reported that patients with SjS with glomerular diseases showed higher mortality rates than those with TIN [5]. Infectious diseases are a common cause of death in patients with SjS showing renal involvement [9]. Thus, although glomerular diseases are less common in patients with SjS, various renal lesions are known to develop. In addition, the treatment strategy differs based on the results of histopathological diagnosis. Therefore, renal biopsy is warranted in patients with SjS with significant urinary abnormalities, such as severe proteinuria and/or hematuria.

In the current case, the patient developed nephrotic syndrome secondary to FSGS. Although secondary FSGS could be attributed to several etiopathogenetic factors such as familial, viral, drug-induced, structural, and functional responses (nephron depletion and hemodynamic changes) [14], this patient showed no obvious findings that could have resulted in secondary FSGS. Therefore, we could not conclusively establish an association between SjS and FSGS. However, a causal association between these diseases and FSGS could be deduced based on the fact that this patient developed both, SjS and PBC (which are rare conditions manifesting with immunological abnormalities), and the observation that immunosuppressive therapy with corticosteroid and cyclosporine was effective in this case.

Unfortunately, there have been no investigations or case reports that concern the prognosis and clinical response of the cases with FSGS and SjS. This is the first case report describing the details of the clinical course and treatment response of the case with FSGS and SjS. Therefore, in order to discuss the appropriate treatment or prognosis, further accumulation of similar cases with current patients is necessary. Because there are few cases of glomerular diseases coexisting with SjS, it is difficult to describe the best treatment options in each glomerular disease. However, we summarized and mentioned the treatment options of each representative glomerular disease developed in the patients with SjS as follows: Concerning the treatment options of the cases with SjS and other glomerular diseases, previous studies have reported the treatment and clinical responses [2–4, 7]. MPGN secondary to cryoglobulinemia develops in the cases with SjS. Cases of MPGN secondary to cryoglobulinemia have been treated with steroids (including pulse therapy), immunosuppressants, rituximab, and plasma exchange therapy [2–4, 7]. In fact, steroids alone or steroid and cyclophosphamide therapy have often achieved complete remission of MPGN cases due to SjS-associated cryoglobulinemia [2–4, 7]. Recently, several reports have suggested that rituximab is effective in MPGN and vasculitis due to cryoglobulinemia and systemic manifestation due to SjS [4, 15, 16]. Mesangial proliferative glomerulonephritis in patients with SjS has been treated with steroids alone or steroids and immunosuppressants (methotrexate, cyclophosphamide, or azathioprine) [2, 4, 17]. Membranous nephropathy in patients with SjS has been treated with steroids alone or steroids and immunosuppressants (cyclosporine, cyclophosphamide, and rituximab) [2, 4, 18]. Minimal change disease was successively treated with steroids alone, and another patient was treated with hydroxychloroquine [3, 4, 19]. Although the clinical response to these treatments has been reported to be successful, as these results were cited from case reports and series studies, there may be a publication bias. Clinical studies will be needed to determine the efficacy of various immunosuppressive therapies against glomerular diseases with SjS.

We report this rare case of SjS and PBC complicated by nephrotic syndrome secondary to FSGS. A greater number of case reports need to be added to the literature to establish the treatment response or renal prognosis of glomerular diseases based on the histopathological diagnosis of renal biopsy specimens.

## Figures and Tables

**Table 1:** Main laboratory data of the current case.

*Blood analysis*					
WBC	8190	/*μ*L	C3	67	mg/dL
RBC	4100000	/*μ*L	C4	15	mg/dL
Hb	12.3	g/dL	CH50	34.1	U/mL
Plt	208000	/*μ*L	CRP	0.43	mg/dL
TP	6.3	g/dL	IgG	1440	mg/dL
ALB	1.7	g/dL	IgA	415	mg/dL
ChE	369	U/L	IgM	1565	mg/dL
T.Ch	323	mg/dL	Cryoglobulins	(–)	
TG	233	mg/dL	Serum immunofixation M protein	(–)	
AST	65	U/L	pH	7.48	
ALT	33	U/L	PaCO2	30.3	mmHg
LDH	392	U/L	PaO2	59.8	mmHg
ALP	1235	U/L	Bicarbonate	22.3	mmol/L
*γ*-GT	664	U/L	Osmolarity	292	mOsm/kg
BUN	14.9	mg/dL	Anti-nuclear antibody	2560	(speckled)
Cr	0.65	mg/dL	Anti-mitochondria M2 antibody	20.6 (+)	
UA	5.7	mg/dL	Anti-double stranded DNA antibody	(–)	
Na	140	mmol/L	Anti-ribonucleoprotein antibody	(–)	
K	3.9	mmol/L	Anti-SS-A/Ro antibody	(–)	
Cl	110	mmol/L	Anti-SS-B/La antibody	(–)	
Ca	7.5	mg/dL	Anti-scleroderma-70 antibody	(–)	
iP	3.7	mg/dL	Anti-Jo-1 antibody	(–)	
Mg	2.5	mg/dL	Anti-centromere antibody	(–)	

*Urinalysis*					
pH	6.0		UUA	62.6	mg/dL
UP	(3+)		UNa	39	mEq/L
OB	(–)		UK	34	mEq/L
NAG	129.3	U/L	UCl	38	mEq/L
*β*2-MG	2539	U/L	UCa	1.0	mg/dL
Osmolarity	408	mOsm/kg	UiP	47.6	mg/mL
UUN	579.3	mg/dL			
UCr	116.9	mg/dL			

WBC: white blood cells; RBC: red blood cells; Hb: hemoglobin; Plt: platelet; TP: total protein; ALB: albumin; ChE: cholinesterase; T.Ch: total cholesterol; TG: triglycerides; AST: aspartate aminotransferase; ALT: alanine aminotransferase; LDH: lactate dehydrogenase; ALP: alkaline phosphatase; *γ*-GT: gamma-glutamyltransferase; BUN: blood urea nitrogen; Cr: creatinine; UA: uric acid; Na: sodium; K: potassium; Cl: chloride; Ca: calcium; iP: inorganic phosphorus; Mg: magnesium; C3: complement 3; C4: complement 4; CH50: 50% hemolytic complement activity; CRP: C-reactive protein; IgG: immunoglobulin G; IgA: immunoglobulin A; IgM: immunoglobulin M; UP: urinary protein; OB: occult blood; NAG: *N*-acetyl-beta-D-glucosaminidase; *β*2-MG: beta-2-microglobulin; UUN: urine urea nitrogen; UCr: urine creatinine; UUA: urine uric acid; UNa: urine sodium; UK: urine potassium; UCl: urine chloride; UCa: urine calcium; UiP: urine inorganic phosphorus.

**Table 2:** Summary of renal biopsy findings of previous studies.

	Renal biopsy findings and frequency (positive biopsies/total biopsies)					
	TIN	MesGN	MN	MPGN	FSGS	MCD
[8]	29/35	2/35	2/35	ND	ND	ND
	(82.9%)	(5.7%)	(5.7%)			
[3]	17/24	ND	1/24	2/24	2/24	ND
	(70.8%)		(4.2%)	(8.3%)	(8.3%)	
[2]	13/35	7/35	2/35	10/35	1/35	ND
	(37.1%)	(20.0%)	(5.7%)	(28.6%)	(2.9%)	
[9]	37/64	21/64	10/64	ND	1/64	ND
	(57.8%)	(32.8%)	(15.6%)		(1.6%)	
[10]	93/95	ND	4/95	8/95	5/95	2/95
	(97.9%)		(4.2%)	(8.4%)	(5.3%)	(2.1%)
[11]	9/17	3/17	ND	ND	1/17	ND
	(52.9%)	(17.6%)			(5.9%)	
[5]	13/25	1/25	1/25	6/25	ND	1/25
	(52.0%)	(4.0%)	(4.0%)	(24.0%)		(4.0%)
[6]	53/103	6/103	37/103	ND	3/103	4/103
	(51.5%)	(5.8%)	(35.9%)		(2.9%)	(3.9%)
[7]	33/41	2/41	1/41	3/41	2/41	ND
	(80.5%)	(4.9%)	(2.4%)	(7.3%)	(4.9%)	

FSGS: focal segmental glomerulosclerosis; MCD: minimal change disease; MesGN: mesangial proliferative glomerulonephritis; MN: membranous nephropathy; MPGN: membranoproliferative glomerulonephritis; ND: no data; TIN: tubulointerstitial nephritis.
